# Supplementary material for: Biomaterial Inks from Peptide-Functionalized Silk Fibers for 3D Printing of Futuristic Wound-Healing and Sensing Materials
Source: Int J Mol Sci. 2023 Jan 4;24(2):947. doi: 10.3390/ijms24020947 (PMC9864705; doi:10.3390/ijms24020947)
Supplement: Supplementary file 1 [file ijms-24-00947-s001.zip › ijms-2136231-supplementary.pdf]

Supporting Information

Biomaterial Inks from Peptide-Functionalized Silk Fibers for 3D Printing of Futuristic Wound-Healing and Sensing Materials

Maria Rachele Ceccarini [1], Valentina Palazzi [2], Raffaele Salvati [2], Irene Chiesa [3], Carmelo De Maria [3], Stefania Bonafoni [2], Paolo Mezzanotte [2], Michela Codini [1], Lorenzo Pacini [4], Fosca Errante [5], Paolo Rovero [5], Antonino Morabito [6], Tommaso Beccari [1], Luca Roselli [2] and Luca Valentini [7,*]

[1] Department of Pharmaceutical Sciences, University of Perugia, 06123 Perugia, Italy
[2] Department of Engineering, University of Perugia, 06125 Perugia, Italy
[3] Department of Ingegneria dell'Informazione and Research Center E. Piaggio, University of Pisa, Largo Lucio Lazzarino 1, 56122 Pisa, Italy
[4] Interdepartmental Research Unit of Peptide and Protein Chemistry and Biology, Department of Chemistry "Ugo Schiff", University of Florence, 50019 Sesto Fiorentino, Italy
[5] Interdepartmental Research Unit of Peptide and Protein Chemistry and Biology, Department of NEUROFARBA, University of Florence, 50019 Sesto Fiorentino, Italy
[6] Dipartimento Neuroscienze, Psicologia, Area del Farmaco e Salute del Bambino NEUROFARBA, Università degli Studi di Firenze, Viale Pieraccini 6, 50121 Firenze, Italy
[7] Civil and Environmental Engineering Department, University of Perugia, Strada di Pentima 4, 05100 Terni, Italy
* Correspondence: luca.valentini@unipg.it; Tel.: +39-0744492924

Supporting Information

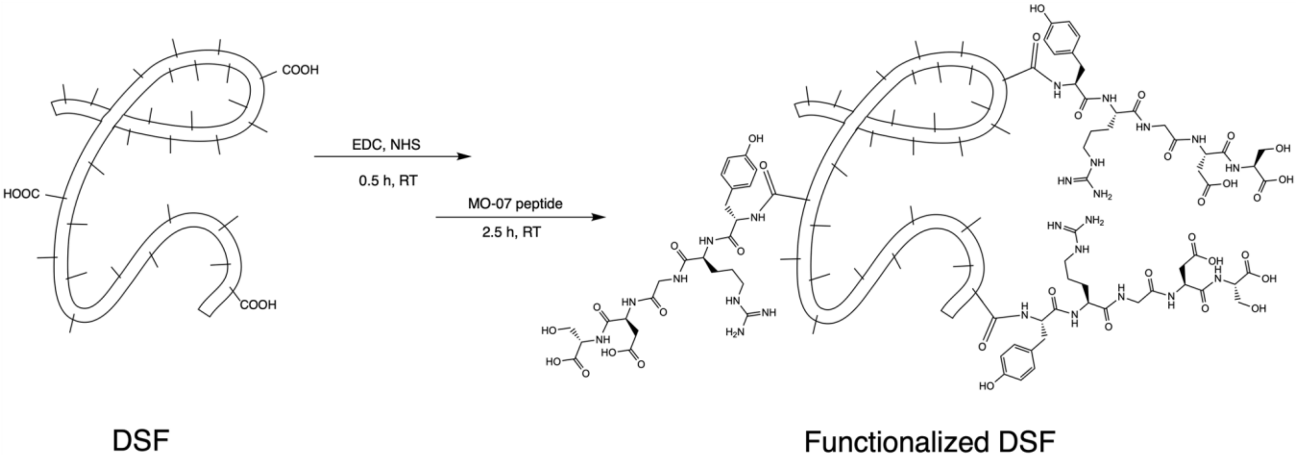

Figure S1. Scheme showing the synthetic pathway for activating -COOH residues on DSF and their reaction with the N-terminal amino group of MO-07 peptide.

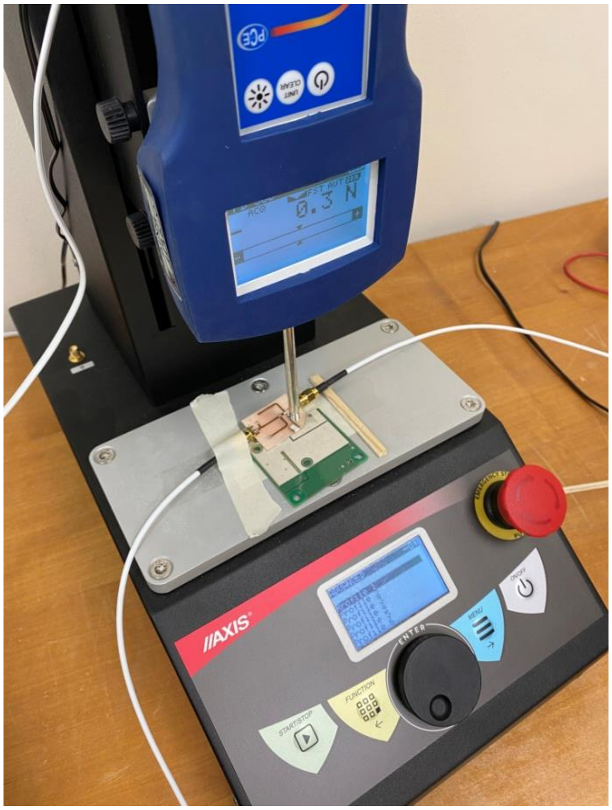

Figure S2. Photo of the dynamometer (model PCE-FB 500) connected to an automated vertical stand used for the piezoresistive measurements.

Supporting Information

S1

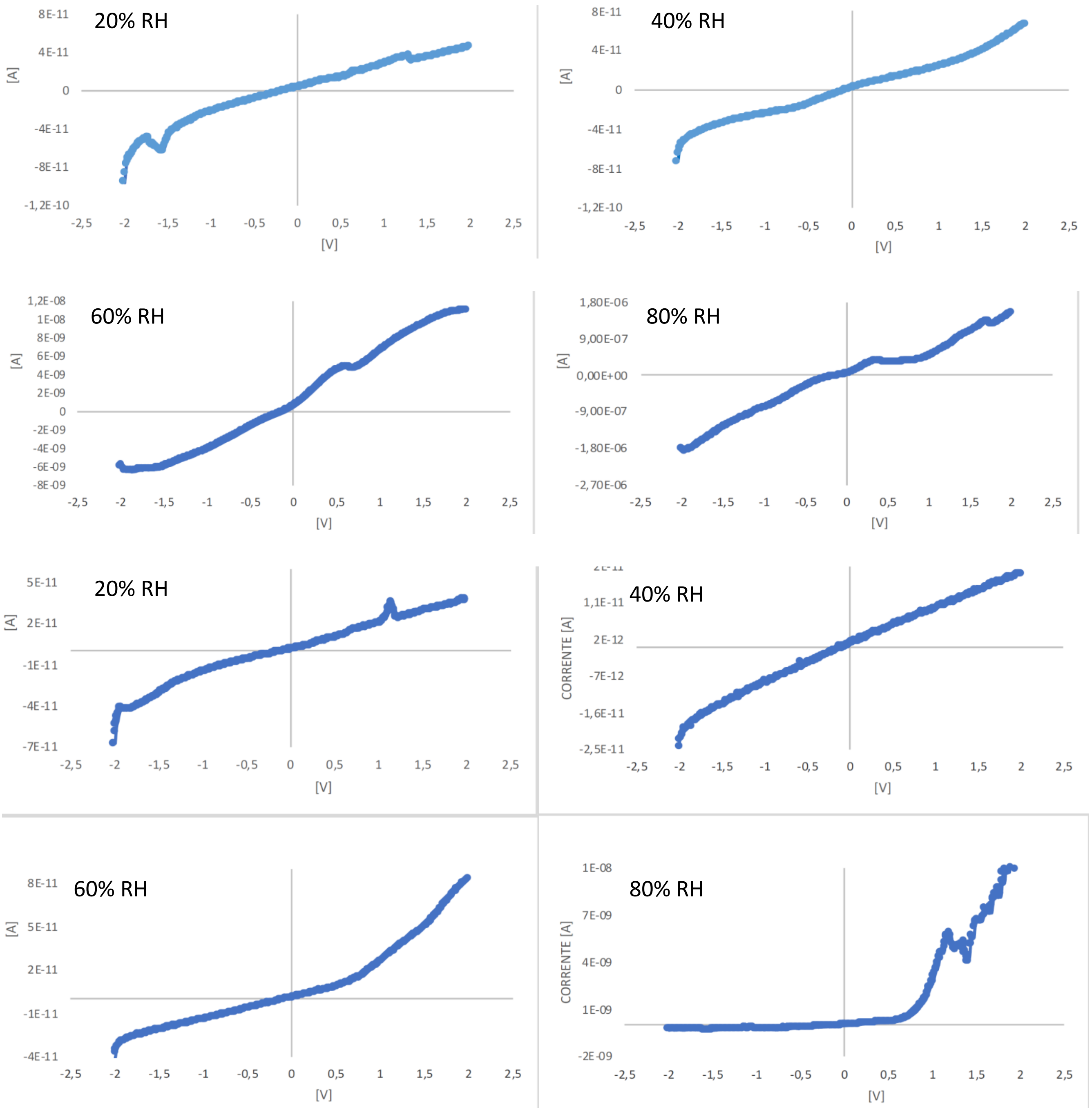

Figure S3. Current-voltage characteristics of (top) RS and (bottom) MO-07/RS 3D printed grids exposed to different values of RH.
